# Molecular Markers and Marker-Assisted Selection Provide Genetic Insights for Identifying Key Quantitative Trait Locus for Watermelon Rind Thickness

**DOI:** 10.3390/ijms251910341

**Published:** 2024-09-26

**Authors:** Zhengxiang Zhao, Shuang Pei, Yuying Song, Tiantian Yang, Yuan Gao, Hao Chai, Feishi Luan, Zicheng Zhu, Xuezheng Wang

**Affiliations:** 1 College of Horticulture and Landscape Architecture, Northeast Agricultural University, Harbin 150030, China; zzxneau@163.com (Z.Z.); ps18804607822@163.com (S.P.); 18845841021@163.com (Y.S.); yttneau2021@gmail.com (T.Y.); 13359860637@163.com (Y.G.); 18234558902@163.com (H.C.); luanfeishi@neau.edu.cn (F.L.); 2 Key Laboratory of Biology and Genetic Improvement of Horticulture Crops (Northeast Region), Ministry of Agriculture and Rural Affairs, Harbin 150030, China

**Keywords:** watermelon, rind thickness, molecular marker, QTL, marker-assisted selection

## Abstract

Rind thickness (RT) is an important agronomic trait in watermelon [*Citrullus lanatus* (Thunb.) Mansf.] and affects watermelon storability. However, genetic studies on this trait, as well as gene regulation studies, are scarce and of limited production significance. We constructed a temporary F_2_ generation using the highly differentiated thick-rind watermelon ‘XiaoXiGua-4’ and the thin-rind watermelon ‘DuanMan’ as parents and localized the *Cla97C02G044120* gene, which controls the thickness of watermelon rind, to the intervals of chromosome 2, CL2-32303995 and CL2-32316840, through 2 years of genetic analysis. No exonic mutations were found in this gene, but two promoter mutations resulted in changes in the promoter progenitor. Fluorescence quantitative PCR analysis revealed highly significant differences in expression at 1 d and 28 d, and the expression was significantly lower in thick-skinned watermelon varieties. Marker-assisted selection (MAS) for this trait was performed using the Caps marker CL2-32303995 and the InDel marker CL2-32316840, which not only verified the stability of the localization interval but also distinguished thick rind from thin rind. These results can be used for germplasm resource screening and have strong breeding significance.

## 1. Introduction

Watermelon [*Citrullus lanatus* (Thunb.) Mansf.] is an annual fruit-type vegetable crop native to Africa. It belongs to the *Cucurbitaceae* family and is very important for the economic development of China. Increasing watermelon yield while improving fruit quality is an important goal for watermelon breeding [1]. It is widely recognized that the ovary wall and receptacle together develop into the rind of the watermelon. This rind is the epidermis of the plant fruit and functions as a natural barrier against the adverse external environment. The rind mainly consists of the exocarp, mesocarp, and endocarp [2]. Moreover, watermelon rinds are divided into outer epidermis, exocarp, mesocarp, and stone cell layer (cluster) structures (which are located between the exocarp and mesocarp) [3]. The rind of melon vegetables has a cuticle and stomata and generally consists of about two layers of cells; the mesocarp consists of relatively large, thin-walled cells; the stone cell layer, or stone cells, is generally recognized to be associated with the rind’s resistance to cracking; and the endocarp cells are very small [4]. Notably, watermelon rind thickness (RT) and hardness determine the edible proportion of the fruit and its storability [5].

The thickness of the rind of a fruit is influenced by several factors. It is generally recognized that RT is often closely related to cell division as well as expansion, with structural genes that regulate cell expansion playing important roles [4]. Undoubtedly, genetic factors are the most important contributors to the differences in the process of rind formation. Many scholars have reported that RT is an important quantitatively inherited trait, which means that genetic factors are the key regulators of these differences, as described in detail later in this article. Moreover, environmental factors such as light, moisture, temperature, and fertilizer also influence RT, and changes in these conditions can also influence differences in RT.

For the time being, the study of watermelon RT is still underdeveloped. Among them, there are scholars who analyzed the genetic law related to the thickness and hardness of watermelon rind by constructing a six-generation population [5]. The performance of the continuous distribution and the analysis revealed the existence of the main effect of gene control on RT and defined genetic characteristics for the obvious quantitative traits. Yang. [3] (2021) through the construction of 133 populations of F_2_ generations of temporary populations, localized a Minor-Effect QTL (RTH2) on chromosome 2 and concluded that it was associated with the thickness of watermelon rind, with a contribution rate of 14.74% and an additive effect of −0.20, which was presumed to be inherited from the parent material “Thin Skin 812”.

A genome-wide association study (GWAS) uncovered valuable insights into the natural variation and phenotypic correlations in RT. Five important SNP loci on chromosome 2 were discovered, i.e., 32344170, 32321308, 32304738, 32328501, and 32311192, which were closely associated with RT through GWAS and were similar to the results of the above studies [6]. Moreover, Fan et al. [7] (2000) by constructing a provisional population of 118 populations of F_2_ generations, localized one primary QTL on each of the No. 1 (qRT1) and No. 3 (qRT3) linkage populations using approaches such as RAPD, SSR, isozyme markers, and morphology markers; Cheng [8] (2015), by using a self-recombinant population of hybrid progeny, F_2_R_7_, obtained four fruit-thickness-related QTLs located in Cluster 3 (two), Cluster 7, and Cluster 10; Sandlin et al. [9] (2012) localized QTL loci related to RT on Cluster 2, Cluster 6, and Cluster 9 (three) by integrating three genetic maps; and Ren et al. [10] (2014) localized QTL loci related to RT on chromosomes 2 (RTH2-1), 5 (RTH5), and 6 (RTH6) by integrating four genetic maps, but their accuracy is still questionable. Loci related to RT in tomato [11], pumpkin [12], winter squash [13], chili pepper [14,15], glutinous maize [16], and sweet maize [17] have also been reported.

Currently, there are no reports of gene regulation for watermelon RT, but many studies on tomato have provided a reference for future research. Czerednik et al. [18] (2015) reported that CDKA1, which regulates the size of tomato cells via overexpression, results in a change in RT. Swinnen et al. [19] (2022) suggested that the absence of the functions of SI KIX8 and SI KIX9 caused the expansion of tomato cells, thereby thickening the rind. De Jong et al. [20] (2009) suggested that SI ARF7 reduced cell expansion at the mRNA level in transgenic tomatoes before thickening the rind. Su et al. [21] (2014) suggested that an RNA interference (RNAi) strategy to silence Sl-IAA17 produced larger fruits than did wild-type tomato, and combined with histological analysis of the fruiting organs, this phenotypic difference was associated with a thicker rind. Conversely, Zhu et al. [22] (2019) and Musseau et al. [23] (2020) suggested that silencing of the b HLH family transcription factor gene Sl PRE2 as well as mutations in the Sl GBP1 gene resulted in thinner RT in tomato. Moreover, studies of RT in crops such as lychee [24] and maize [25] have also been reported.

In melon, it has been reported that RT has a significant effect on yield [26], but this effect has not been reported in watermelon. Gong et al. [4] (2022) suggested that watermelons with thicker rinds result in fewer edible parts, and the flavor is affected. In tomato [27], the same conclusion was reached: the thicker the exocarp is, the greater the resistance to storage and transportation. Therefore, the thickness of watermelon rind has a certain influence on consumer purchase, and clarifying the genetic mechanism of watermelon RT is important for improving watermelon storage and transportation resistance resources. In this study, two watermelon inbred lines with large differences in RT were used as parents to formulate an F_2_ genetic population, and corresponding cleaved amplified polymorphic sequence (Caps), insertion–deletion (InDel), and kompetitive allele-specific PCR (KASP) markers were used to draw genetic linkage maps and conduct QTL analyses for watermelon RT traits, as well as to validate the stability of the QTLs obtained in different environments and to develop molecular markers for distinguishing between thick- and thin-rind watermelons. The results of this study are aimed at providing theoretical and practical guidance on the fine localization of the genes associated with RT traits and aiding in the construction of a theoretical basis and application reference for the accurate identification of watermelon RT trait genes.

## 2. Results

### 2.1. Genetic and Phenotypic Characteristics of Watermelon RTs

**Figure 1:**
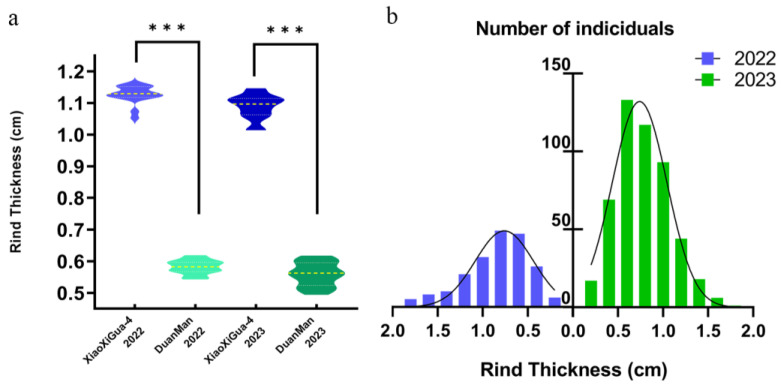
Analysis of rind thickness (RT) in watermelon: (**a**) Parental RT traits. Note: *** indicates a significant difference at the 0.001 level; (**b**) Histogram of frequency distribution of watermelon RT for F_2_ in 2022 and 2023.

The RTs of 16 thick-rind ‘XiaoXiGua-4’ watermelons and 12 thin-rind ‘DuanMan’ watermelons were measured in 2022, and the RTs of 15 thick-rind ‘XiaoXiGua-4’ watermelons and 17 thin-rind ‘DuanMan’ watermelons were measured in 2023. The data of the above parents were analyzed using independent sample *t* tests in the SPSS v.20.0 software (Figure 1a). The RTs of ‘XiaoXiGua-4’ were 1.13 ± 0.03 cm and 1.09 ± 0.04 cm in 2022 and 2023, respectively, and the RTs of ‘DuanMan’ were 0.58 ± 0.02 cm and 0.56 ± 0.04 cm, respectively. In both years of data, the RT of the ‘XiaoXiGua-4’ material was highly significantly greater than that of the ‘DuanMan’ material (*p* < 0.001).

An analysis of watermelon RT data from the segregating population of the F_2_ generation yielded a total of 204 valid data points in 2022 and 498 in 2023 (Figure 1b). The frequency distribution conformed to a normal and continuous distribution and reflected the quantitative genetic characteristics.

### 2.2. Genetic Linkage Analysis Placed the RT Locus into a 12.845 kb Region on Chromosome 2

First, high-quality genomic DNA was extracted (Supplementary Figure S1). A total of 47 pairs of Caps primers were designed, and 45 pairs had polymorphisms, with a polymorphism rate of 95.74%. In addition, twenty pairs of InDel primers were designed, and seven pairs had polymorphisms, with a polymorphism rate of 35%; Finally, eight pairs of KASP primers were designed, and eight pairs had polymorphisms, with a polymorphism rate of 100% (Supplementary Figure S2). With these primers, P_1_, P_2_, and F_1_ generations with different bp lengths could be distinguished and could be used for genotyping (Supplementary Figure S3).

**Figure 2:**
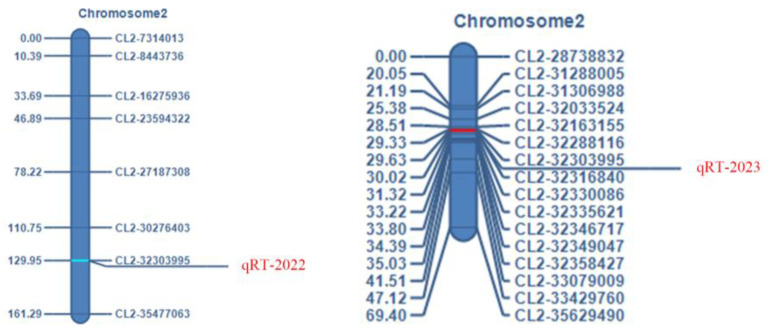
Genetic linkage mapping.

Based on the genotyping results, combined with the team’s published genetic linkage mapping results (reference sequence 97103 v1, http://cucurbitgenomics.org/organism/1 (accessed on 2 April 2021)) [28], a genetic linkage map of watermelon chromosome 2 (reference sequence 97103 v2, http://cucurbitgenomics.org/organism/21 (accessed on 20 December 2023)) was constructed (Figure 2). A total of 250 F_2_ generation populations in 2022 localized the main effect QTL of watermelon RT within the intervals CL2-32303995 and CL2-35477063 on chromosome 2, with an interval size of 3.17 Mb, a logarithm of odds (LOD) value of 4.5833, a contribution rate of 11.1790%, an additive effect with an additive effect value of 1.6376, and a negative dominant effect with a dominant effect value of −0.2315. A total of 527 F_2_ generation populations in 2023 localized the main QTL for watermelon RT within the intervals CL2-32303995 and CL2-32316840 on chromosome 2, with an interval size of 12.85 kb, a LOD value of 7.1057, a contribution of 6.6129%, an additive effect with an additive effect value of 0.9829, and a negative dominant effect with a dominant effect value of −0.5641.

### 2.3. Analysis of the Candidate Gene Cla97C02G044120

**Figure 3:**
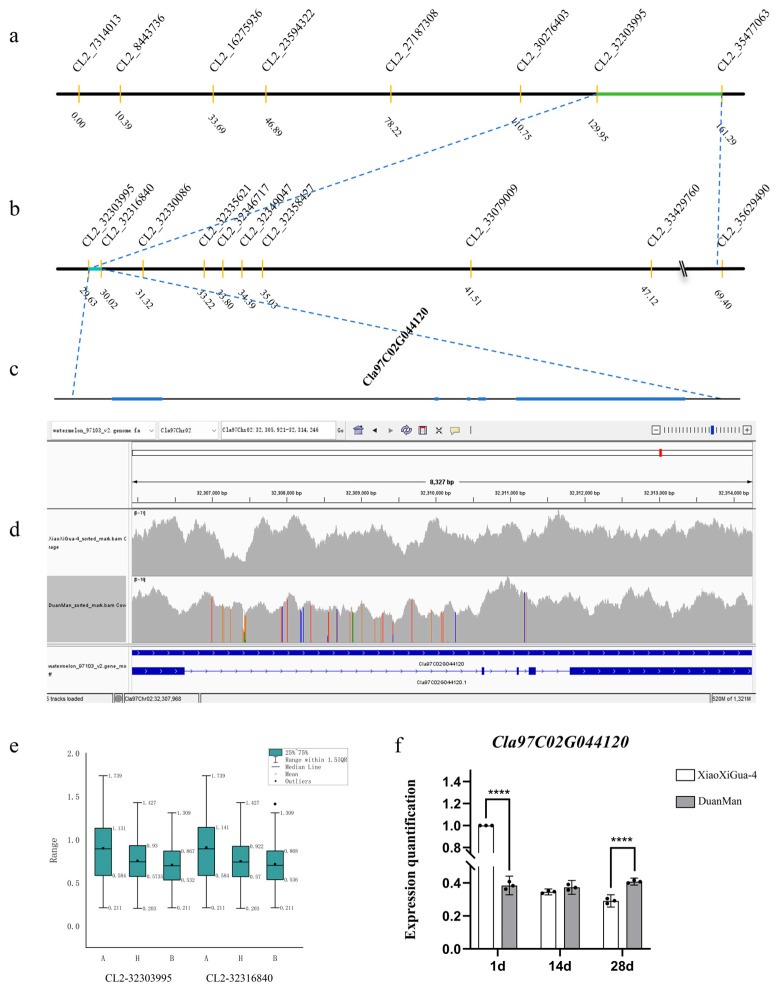
QTL localization of RT in watermelon and the analysis of candidate genes: (**a**) QTL localization results for 2022; (**b**) QTL localization results for 2023; (**c**) Gene structure within the localization interval; (**d**) Base mutations in candidate genes between XiaoXiGua-4 and DuanMan; (**e**) Distribution of phenotypic data among markers in 2023; (**f**) Candidate gene expression analysis at different time periods. Note: **** indicates a significant difference at the 0.0001 level.

The primary QTL for RT in watermelon was localized in the intervals CL2-32303995 and CL2-32316840 on chromosome 2, totaling 12.85 kb, using a year-by-year step-by-step approach (Figure 3a–c). There was only one gene in the interval, which was *Cla97C02G044120*, based on the annotation of the watermelon reference genome 97103 v2 (http://cucurbitgenomics.org/organism/21 (accessed on 20 December 2023)), annotated as elongation factor 2; its position on chromosome 2 was Cla97Chr02:32305922..32314246. An analysis and comparison of the two parental materials revealed a total of 36 intronic mutations (Supplementary Figure S4) and 27 insertion deletions, but no exonic mutations; the protein structure did not change (Figure 3d).

The total number of mutations at the predicted promoter was 10. The promoter of the gene (2000 bp upstream of the gene) was analyzed using Plant CARE (https://bioinformatics.psb.ugent.be/webtools/plantcare/html/ (accessed on 20 December 2023)), and two mutations were predicted to cause changes in promoter elements. The first one was 25 bp within the promoter of the gene, where a C base of the parent genome was mutated to a T base, resulting in a change from the presence to the absence of the promoter element CAAT-box; the second mutation was 1083 bp within the promoter of the gene, where a C base of the parent genome was mutated to an A base, resulting in a change from the absence to the presence of the promoter element CTAG-motif.

Based on the “Peak Up and Peak Down” diagram in the QTL IciMapping v4.1.0.0 software, the localization interval was extended to the intervals of CL2-32163155 and CL2-32335621 on chromosome 2, totaling 172.47 kb. Twenty-one genes were identified within the intervals, including *Cla97C02G044120* (Table 1). Of these, *Cla97C02G043940* had one exonic mutation, *Cla97C02G044100* had one exonic mutation, and *Cla97C02G044130* had four exonic mutations, respectively; the remaining genes had no exonic mutations (Supplementary Figure S5). However, none of the exonic mutations were predicted to result in changes in the protein structure. Mutations in the predicted promoter (2000 bp upstream of the gene) resulted in a greater number of changes in promoter elements (Table 2).

The results of real-time fluorescence quantitative PCR (qRT-PCR) analysis of cDNA from the rind of watermelon at different periods showed that there was a highly significant difference in the expression at the 1 d and 28 d periods, and the expression in thick-skinned watermelon varieties was significantly lower, considering that this gene may increase the RT of watermelon and that with the development of fruits, the expression of this gene gradually decreases, resulting in a thinner rind (Figure 3f); however, we still need further evidence to demonstrate how *Cla97C02G044120* regulates the RT of watermelon.

### 2.4. Marker-Assisted Selection (MAS) of the RT Trait in a Natural Watermelon Panel

**Figure 4:**
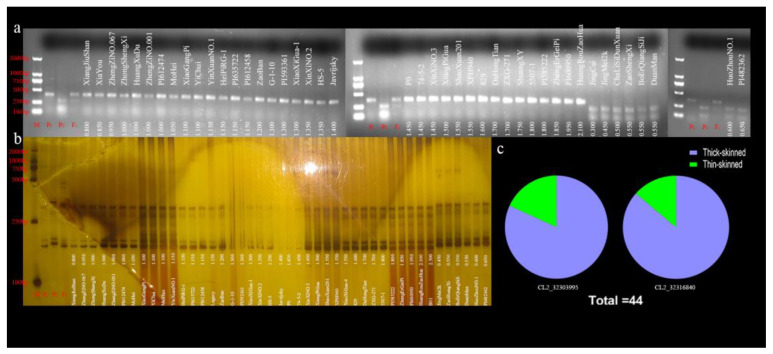
Validation of molecular markers’ natural variety in populations: (**a**) Caps marker; (**b**) InDel marker; (**c**) The distribution of thick-rind versus thin-rind watermelon in the two molecular markers.

To determine whether these markers could be applied in RT molecular selection, we chose 44 watermelon accessions (36 thick rinds and 8 thin rinds in Supplementary Table S1) to determine the correlation between genotype and phenotype with the Caps marker CL2-32303995. The thick-rind watermelon accessions presented 375 bp bands, whereas the lanes for accessions with thin rinds lacked these 375 bp bands (Figure 4a). The results showed that this marker can distinguish plants with thick rinds from those with thin rinds.

We chose 44 watermelon accessions (38 thick rinds and 6 thin rinds, as shown in Supplementary Table S1) to determine the correlation with the InDel marker CL2-32316840. The thick-rind watermelon accessions presented 164 bp bands, whereas the lanes for accessions with thin rinds did not show the 169 bp bands (Figure 4b).

The results indicated that the markers could distinguish plants with thick rinds from those with thin rinds. The consistency between genotypes and phenotypes was 100%. At the same time, the distribution of thick-rind versus thin-rind watermelon in the two molecular markers is also listed (Figure 4c).

## 3. Discussion

Zhan et al. [29] (2020) suggested that the dense arrangement of cells, along with the formation of a well-defined cell cluster structure, was responsible for the production of thick rinds in watermelon, whereas the thin rinds in other watermelon varieties were due to the relatively longer cells of their epidermal tissues, with larger cells in the exocarp and mesocarp, which was similar to the findings of Du et al. [30] (2012) in cucumber. Gao et al. [31] (2013) suggested that rounded rind cells, lateral growth of cells on the cuticle, continuous straight bands of xylem, and larger clusters of stone cells were responsible for thick rinds in watermelon. Liu et al. [32] (2012) compared common watermelon with diploid watermelon and reported that the structure of the rind of polyploid watermelon was different from that of common watermelon, leading to an influence on the thickness of the rind. Guan et al. [33] (2010) reported that the structure of the exocarp was related to the number of cell layers and the thickness of the cell wall. In contrast, in sorghum, the structure with the greatest influence on the thickness of the rind was the mesocarp [34].

Wang et al. [35] (2020) reported that rind hardness was highly significantly positively correlated with RT, with a correlation coefficient of 0.99. Through genetic analysis of RT in watermelon, it was reported that RT was normally distributed, which is a typical quantitative trait; through genetic modeling analysis, it was determined to be a typical quantitative trait controlled by multiple genes [5,8]. Zhu et al. [36] (2017) reported a highly significant positive correlation between RT and plot yield. Wang et al. [26] (2016) reported the same results in melon, and multiple regression analysis revealed that this correlation reached a highly significant level, while its normal distribution was also reported [37]. Similarly, in tomato, the RT of the natural population was normally distributed [11], whereas Yang et al. [27] (2019) took a different perspective and reported that the thicker the exocarp of processed tomatoes is, the lower the decay rate of the processed tomatoes after they have been left for a period of time. In a study on citrus plants, Li et al. [38] (2021) reported that after flower shedding, the thickness of the rind increases while the fruit develops, reaching a peak of 0.50 cm at 45 d of full bloom, and then gradually decreases to approximately 0.19 cm; however, in glutinous maize [39], the thickness of the rind during the period of grouting shows a parabolic dynamic change, reaching a peak after 20 d of pollination and then gradually thinning [40]. This phenomenon has not been reported in watermelon.

Moreover, it is widely recognized that the RT of watermelon is closely related to the storage period, storage resistance, transportation resistance, and cracking of the fruit [41,42]. At present, there have been few gene localization studies on watermelon rind, for which the field is relatively sparse. Moreover, there have been no reports on the gene regulation of RT in watermelon.

Previous forward genetics studies utilizing resequencing and genotyping have facilitated gene mapping and research on many complex traits [43]. In this study, molecular marker technology (Caps, InDel, KASP) was used to locate the gene that controls watermelon RT between markers Chr02_32303995 and Chr02_32316840 on chromosome 2 (approximately 12.845 kb), revealing that the region contained one predictive gene. Gong et al. [6] (2022) identified five SNP loci on chromosome 2 of watermelon, namely, 32344170, 32321308, 32304738, 32328501, and 32311192, which were closely associated with RT through GWAS and were similar to the results of the above studies. Our findings are similar to the results of their studies. There are differences in approach and direction; therefore, it is also a good example of the accuracy of the conclusions produced by two different experiments. We constructed a temporary population of the F_2_ generation and then used it to localize the watermelon rind thickness. We performed an auxiliary validation using more natural population data, which was 100 per cent consistent with our localization results. Furthermore, we have developed relevant molecular markers that make it possible to screen for this trait at the seedling stage at low cost.

To our knowledge, this study provides the report of a gene influencing watermelon RT. Although there were no mutations in the coding region of the candidate gene, we detected two SNPs mutations in the predicted promoter region that changed the cis-acting elements of the candidate gene *Cla97C02G044120*. The qRT-PCR results revealed that the relative expression of *Cla97C02G044120* was greater in thin-rind watermelon plants than in thick-rind watermelon plants. Considering the possibility that the expression of this gene increases the RT of watermelon, as the fruit develops, the expression of the gene might progressively decrease, making the RT thinner. We will continue to refine whether the expression of different parts of this gene have any effect on the thickness of the rind in subsequent studies.

The MAS for this trait was constructed in this study, and molecular MAS using the Caps marker CL2-32303995 and the InDel marker CL2-32316840 distinguished thicker rinds from thinner rinds and could be used to screen germplasm resources, with strong breeding significance.

## 4. Materials and Methods

### 4.1. Plant Materials and Genetic Mapping Population

**Figure 5:**
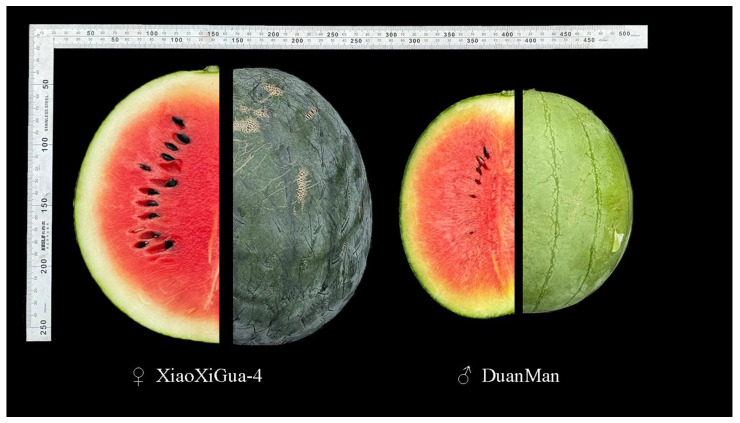
Phenotypes of the parental line RT.

The experimental parents ‘XiaoXiGua-4’ and ‘DuanMan’ were obtained from the Watermelon and Melon Molecular Genetic Breeding Laboratory, Northeast Agricultural University (Figure 5). Using the thicker-skinned variety ‘XiaoXiGua-4’ as the female parent and the thinner-skinned variety ‘DuanMan’ as the male parent, the F_1_ generation was bred by crossbreeding (Supplementary Table S2), and the closest to the mean of the F_1_ generation seeds were self-fertilized to obtain the F_2_ generation population. The F_2_ population was used to perform genetic mapping. Moreover, natural population material from 48 varieties of watermelon was grown for MAS validation, as shown in Supplementary Table S1.

For genetic map construction, segregation analysis and MAS validation, the F_2_ population was grown together with the parental lines: parental and F_1_ generation materials were planted with 20 plants per year; 250 F_2_ were planted and harvested in the summer of 2022 (Harbin, greenhouse); 527 F_2_ were planted in 2023 (Harbin, greenhouse); and a natural population of 48 varieties of watermelon was planted in 2023 (Harbin, greenhouse).

Conventional field management methods were used, with double-twisting of the vines and leaving one watermelon per plant after fruit set. When the fruits were ripe (approximately 32 d after pollination), they were harvested for the next trait investigation. Each watermelon was cut longitudinally along the center, and the thickness of the watermelon rind was measured at an angle of 120 degrees along the center of the cut surface via Vernier calipers to obtain the average values of the three parts of the watermelon rind, the measurements of which were accurate to 0.001 cm.

### 4.2. DNA and RNA Extraction

Young leaves from the two parental lines and the natural community were collected and stored at −80 °C. A modified CTAB method was used for genomic DNA extraction [44], as described by Gao [45] (2018) in his PhD thesis. DNA was evaluated by electrophoresis in a 1.0% agarose gel.

RTs of the two parental lines were collected at 1, 14, and 28 d after pollination and stored at −80 °C. RNA was extracted using a Total RNA Extractor (Shanghai Sangyo) and reverse transcribed with ReverTra Ace^®^ qPCR RT Master Mix with gDNA Remover (Toyobo). The related procedures were carried out with reference to the product manual.

### 4.3. Molecular Marker Development and Genetic Mapping

The resequenced data were compared with the available watermelon reference genome (97103 v2 Genome) from the Cucurbitaceae Official Site (http://cucurbitgenomics.org/organism/21 (accessed on 20 December 2023)) to identify reliable SNPs through a filter pipeline. To narrow down candidate regions and verify the accuracy of the initial localization results of the genetic map, Caps markers were established based on SNPs, and InDel markers and KASP were established based on the resequencing data, as shown in Supplementary Tables S3–S5.

PCR amplification was performed in a 20 µL reaction with 2 µL of DNA, 0.2 µL of Taq polymerase, 0.3 µL of deoxyribonucleoside triphosphates, 2 µL of buffer, 1 µL of 10 µM each primer, and 13.5 µL of double-distilled water. The PCR protocol was performed under the following conditions: initial denaturation at 94 °C for 5 min; 30 cycles at 94 °C for 30 s, 60 °C (reduced by 0.5 °C per cycle) for 30 s, and 72 °C for 45 s; a final extension at 72 °C for 10 min; and storage at 4 °C. The corresponding restriction endonucleases were subsequently used to digest the amplified PCR products for Caps priming at 37 °C for 3.5 h following the product brochure. The digested products were separated on 1.0% agarose gels and visualized. Indel priming was performed by polyacrylamide gel electrophoresis to verify primer polymorphisms. KASP primers were sent to the Vegetable Center of the Beijing Academy of Agricultural and Forestry Sciences for verification of primer polymorphisms using the LGC Genomics typing platform. The markers with polymorphisms were used for fine mapping. Among them, bands consistent with the parent are noted as “A”; bands consistent with the parent are noted as “B”; bands consistent with F_1_ are noted as “H”; and the absence of bands is uniformly noted as “-”. Genetic linkage mapping was constructed by composite interval mapping.

### 4.4. Candidate Gene Prediction and Analysis with MAS System Verification

The sequence and gene function data were retrieved from the Cucurbit Genomics Database (http://cucurbitgenomics.org/ (accessed on 20 December 2023)). Initial targeting was performed on 250 individuals harvested using 8 pairs of Caps primers in 2022, and fine targeting was performed on 527 individuals harvested using 16 pairs of Caps primers, InDel primers, and KASP primers in 2023.

The qRT-PCR was performed on the relevant candidate genes to detect differences in expression. Candidate gene qRT-PCR-specific primers were designed using an online website to design qRT-PCR primers with Cla020175 (ClYLS8) as the internal reference gene. The primers for the internal reference gene were as follows: F: 3′-AGAACGGCTTGTGGTCATTC-5′; R: 3′-GAGGCCAACACTTCATCCAT-5′.

Combined with the localization results, the candidate genes within the localization interval were screened by Integrative Genomics Viewer (IGV) identification of resequencing data based on the data from the reference sequence Watermelon 97103 v2 on the official website of Cucurbitaceae (http://cucurbitgenomics.org/ (accessed on 20 December 2023)), and the related genes were identified.

Caps and InDel markers in fine-mapped regions were used to correlate genotypes and phenotypes by using the genomic DNA of a natural population of 48 varieties of watermelon planted in 2023 for the molecular MAS system verification.

### 4.5. Statistical Analysis

The data were recorded and collated using WPS Office v.8.1.0 software (Kingsoft, Peking, China). Analysis of variance (ANOVA) was used to analyze significant differences. Analyses were performed using SPSS v.20.0 software (SPSS Inc., Chicago, IL, USA). Prism 8.0.2 software (GraphPad Inc., La Jolla, CA, USA) was used to prepare the figures.

## 5. Conclusions

In conclusion, we finely mapped a watermelon rind trait locus on chromosome 2 and identified the most likely candidate gene, *Cla97C02G044120*. The Caps marker CL2-32303995 and the InDel marker CL2-32316840 developed in this study should be useful for selection in marker-assisted breeding programs and may provide a first step for selective breeding and genetic modification to increase the prevalence of this economically important trait. Few studies have been conducted to characterize the molecular markers for RT in watermelon. We used a 2-year F_2_ generation population for QTL localization and validated it using the MAS technique. In future research, we will use gene editing approaches to further verify the candidate genes.

## Figures and Tables

**Table 1:** Candidate genes for the primary QTL for RT.

Gene ID	Description	Position
*Cla97C02G043940*	Ethylene-responsive transcription factor 3-like	32161415 .. 32162074 (+)
*Cla97C02G043950*	Triacylglycerol lipase 2, putative	32171738 .. 32173760 (+)
*Cla97C02G043960*	Lipase	32176692 .. 32179249 (+)
*Cla97C02G043970*	Protein disulfide isomerase-like 2-3	32182402 .. 32186689 (+)
*Cla97C02G043980*	Centromere/kinetochore protein zw10 homolog	32188755 .. 32196057 (−)
*Cla97C02G043990*	Lipid-A-disaccharide synthase	32202021 .. 32205359 (+)
*Cla97C02G044000*	Unknown protein	32206780 .. 32207294 (+)
*Cla97C02G044010*	Unknown protein	32208194 .. 32208541 (+)
*Cla97C02G044020*	Ubiquitin and WLM domain-containing protein	32218483 .. 32221679 (+)
*Cla97C02G044030*	3-oxoacyl-[acyl-carrier-protein] synthase	32223103 .. 32229573 (+)
*Cla97C02G044040*	F-box family protein	32234318 .. 32236622 (−)
*Cla97C02G044050*	Heat stress transcription factor A-4c-like	32238954 .. 32239963 (−)
*Cla97C02G044060*	Transcription elongation factor B polypeptide 3-like	32246890 .. 32248741 (+)
*Cla97C02G044070*	Citrate synthase	32252262 .. 32256301 (−)
*Cla97C02G044080*	Ubiquinone biosynthesis O-methyltransferase, mitochondrial	32264788 .. 32269245 (−)
*Cla97C02G044090*	Leucine-rich repeat receptor-like protein kinase family protein	32278703 .. 32280043 (−)
*Cla97C02G044100*	Serine/threonine protein phosphatase 7 long form	32283295 .. 32286529 (−)
*Cla97C02G044110*	Serine/threonine-protein phosphatase	32290370 .. 32300164 (−)
*Cla97C02G044120*	Elongation factor 2	32305922 .. 32314246 (+)
*Cla97C02G044130*	Protein kinase, putative	32320831 .. 32328837 (+)
*Cla97C02G044140*	Elongation factor 2	32332095 .. 32335291 (+)

**Table 2:** Expanded interval promoter mutation status.

Gene ID	Position	P_1_	P_2_	Name	Organism	Function
*Cla97C02G043940*	1112	A *	T	CAAT-box	Pisum sativum	Common cis-acting element in promoter and enhancer regions
	1112	A	T *	CAAT-box	Nicotiana giutinosa	-
	1688	G	A *	CAAT-box	Nicotiana giutinosa	-
*Cla97C02G044090 ^R^*	793	C *	T	TATA-box	Arabidopsis thaliana	Core promoter element around −30 of transcription start
	1724	G	T *	GARE-motif	Brassica oleracea	Gibberellin-responsive element
*Cla97C02G044100 ^R^*	141	G *	A	CAAT-box	Pisum sativum	Common cis-acting element in promoter and enhancer regions
	393	C *	T	CAAT-box	Nicotiana giutinosa	-
	391	C	T *	Box 4	Petroselinum crispum	Part of a conserved DNA module involved in light responsiveness
	407	T *	A	MRE	Petroselinum crispum	MYB binding site involved in light responsiveness
	410	T	A *	CAAT-box	Pisum sativum	Common cis-acting element in promoter and enhancer regions
*Cla97C02G044110 ^R^*	397	C *	T	CAAT-box	Arabidopsis thaliana	Common cis-acting element in promoter and enhancer regions
	398	C *	T	CAAT-box	Nicotiana giutinosa	-
*Cla97C02G044120*	25	C *	T	CAAT-box	Petunia hybrida	Common cis-acting element in promoter and enhancer regions
	1083	C	A *	CTAG-motif	Avena sativa	-
*Cla97C02G044130*	151	C *	G	MYB-binding site	Nicotiana tabacum	-
	151	A *	T	MYB-binding site	Nicotiana tabacum	-
*Cla97C02G044140*	731	T *	G	CAAT-box	Nicotiana glutinosa	-
	1097	C *	T	Unnamed__4	Petroselinum hortense	-
	1285	T	C *	CAAT-box	Pisum sativum	Common cis-acting element in promoter and enhancer regions

* indicates the presence of a promoter element. ^R^ indicates that the gene is in a reverse orientation.

## Data Availability

Available upon request from the corresponding author(s).

## Supplementary Materials

The following supporting information can be downloaded at: https://www.mdpi.com/article/10.3390/ijms251910341/s1.
