# Supplementary material for: Molecular Markers and Marker-Assisted Selection Provide Genetic Insights for Identifying Key Quantitative Trait Locus for Watermelon Rind Thickness
Source: Int J Mol Sci. 2024 Sep 26;25(19):10341. doi: 10.3390/ijms251910341 (PMC11477180; doi:10.3390/ijms251910341)
Supplement: Supplementary file 1 [file ijms-25-10341-s001.zip › Supplementary Table S1.pdf]

Supplementary Table S1. Phenotypes and genotypes of 48 watermelon varieties

Breed name	Rind thickness (cm)	Molecular diagnosis of Caps	Molecular diagnosis of InDel
B01	2.500	-	A
HuangRouZaoHua	2.100	A	A
PI600950	1.950	A	A
ZhengErGeiPi	1.850	A	A
PI585222	1.800	A	A
5507-1	1.800	A	A
ShuangXY	1.750	A	-
DaHongTian	1.700	A	A
ZXG-271	1.700	A	A
829	1.600	A	A
ShaoXuan201	1.550	A	A
XiaoXiGua-4	1.550	-	A
XPH940	1.550	A	A
XiangPiGua	1.500	A	A
74-5-2	1.450	A	A
P0	1.450	A	A
XinXiNO.3	1.450	A	A
Javrijsky	1.400	A	A
HS-5	1.350	A	A
XinXiNO.2	1.350	A	A
G-1-10	1.300	A	A
PI593361	1.300	A	A
XiaoXiGua-1	1.300	A	A
ZaoBan	1.200	A	A
HeiPiRG-1	1.150	A	A
Legacy	1.150	-	A
PI612458	1.150	A	A
PI635722	1.150	A	A
YinXuanNO.1	1.150	A	A
MeiHao	1.100	-	A
XiaoGangPi	1.100	A	A
YiChui	1.100	A	A
MoHei	1.050	A	A
HuangXuDu	1.000	A	A
PI612474	1.000	A	A
ZhengZiNO.001	1.000	A	A
ZhongShengXi	1.000	A	A
ZhengZiNO.067	0.950	A	A
XiaYou	0.850	A	-
XiangJiuShan	0.800	A	A
PI482362	0.650	B	B

Continued Table S1

Phenotypes and genotypes of 48 watermelon varieties

Breed name	Rind thickness (cm)	Molecular diagnosis of Caps	Molecular diagnosis of InDel
HuoZhouNO.1	0.600	B	B
DuanMan	0.550	B	B
BoErQiangSiJi	0.550	B	B
ZaoShengXi	0.550	B	B
ChaLiSiDunXuan	0.500	B	-
JingMei2k	0.450	B	B
JingCai	0.300	B	-
