# Supplementary material for: Molecular Markers and Marker-Assisted Selection Provide Genetic Insights for Identifying Key Quantitative Trait Locus for Watermelon Rind Thickness
Source: Int J Mol Sci. 2024 Sep 26;25(19):10341. doi: 10.3390/ijms251910341 (PMC11477180; doi:10.3390/ijms251910341)
Supplement: Supplementary file 1 [file ijms-25-10341-s001.zip › Supplementary Table S2.pdf]

Supplementary Table S2. Information on parental and F1 generation rind thickness (cm)

2022				2023			
Rind Thickness	Female (XiaoXiGua-4)	Parent (DuanMan)	F_1	Rind Thickness	Female (XiaoXiGua-4)	Parent (DuanMan)	F_1
1	1.121	0.583	1.103	1	1.104	0.578	1.078
2	1.074	0.597	1.108	2	1.082	0.512	0.983
3	1.153	0.571	1.060	3	1.063	0.547	1.072
4	1.132	0.597	1.106	4	1.088	0.544	1.091
5	1.117	0.619	1.015	5	1.116	0.555	1.053
6	1.129	0.550	1.054	6	1.097	0.568	1.004
7	1.120	0.567	1.039	7	1.108	0.594	1.107
8	1.116	0.595	1.037	8	1.015	0.520	1.012
9	1.130	0.582	1.082	9	1.112	0.596	1.073
10	1.154	0.608	1.053	10	1.111	0.613	0.985
11	1.162	0.542	1.003	11	1.115	0.505	1.020
12	1.053	0.568	1.047	12	1.065	0.494	1.027
13	1.134	-	1.015	13	1.147	0.567	1.094
14	1.124	-	1.007	14	1.139	0.562	0.985
15	1.155	-	-	15	1.052	0.527	1.067
16	1.149	-	-	16	-	0.602	1.075
17	-	-	-	17	-	0.618	-
Average	1.126	0.582	1.052	Average	1.088	0.559	1.045
Standard deviation	1.029	0.023	0.036	Standard deviation	0.040	0.038	0.043
