# Supplementary material for: Molecular Markers and Marker-Assisted Selection Provide Genetic Insights for Identifying Key Quantitative Trait Locus for Watermelon Rind Thickness
Source: Int J Mol Sci. 2024 Sep 26;25(19):10341. doi: 10.3390/ijms251910341 (PMC11477180; doi:10.3390/ijms251910341)
Supplement: Supplementary file 1 [file ijms-25-10341-s001.zip › Supplementary Table S3.pdf]

Supplementary Table S3. Information on Caps markers based on 97103 v2 reference genome on chromosome 2

Position	TM (°C)	Enzyme	Forward Sequence	Reverse sequence
CL2-7314013	57.9	AluI	CACCACCTATGTCTGTAGAGCAATG	CCAAGAGTTTGTGAGGCAAGAAGT
CL2-8443736	55.7	DraI	GCATATCTCGGAATCAATCCTCTAC	TGGTTACTGCTGCTTGACTGT
CL2-16275936	54.9	MspI	GACTCCAACTTCAAGAACATCCA	TTGCCACCTCAAGCCACTT
CL2-23594322	54.2	MboII	GCTTCCATCAAGGCGTGTT	GGGTGTGACATATAGGGTGTTG
CL2-27187308	48.9	HindII	CACATACCAACTTGAGAACA	GCTGAGATAGACCTGACAT
CL2-30276403	54.9	BsaHI	CCATCGTCTTCTTGCTTCATCA	GGATTGTGACTGAGGTTGTTGA
CL2-31288005	51	HindIII	GGATCAGCCAAACATAACTCAC	GGTTGAATCACAACATAAGCAC
CL2-31306988	52.75	MboI	ATAATAGCCCACCACGTTCCT	GGTCATTGGTTCTAGTCTTACATCT
CL2-32163155	54	MspI	CTTGATTCTTCTTGCCACACCAT	GCCTAGCCTAGATTTCCCTTATGT
CL2-32303995	54.4	MspI	GATTCAACGGTCACGACGAA	GCAAGGCTACAAGGACAATCT
CL2-35477063	54.3	MspI	TCTCTGCTAACACCACCTGAT	AAGGGACATCAAAGTCAGTTGT
