# Supplementary material for: Molecular Markers and Marker-Assisted Selection Provide Genetic Insights for Identifying Key Quantitative Trait Locus for Watermelon Rind Thickness
Source: Int J Mol Sci. 2024 Sep 26;25(19):10341. doi: 10.3390/ijms251910341 (PMC11477180; doi:10.3390/ijms251910341)
Supplement: Supplementary file 1 [file ijms-25-10341-s001.zip › Supplementary Table S4.pdf]

Supplementary Table S4. Information on InDel markers based on 97103 v2 reference genome on chromosome 2

Position	TM (°C)	Forward Sequence	Reverse sequence
CL2-28738832	52.25	GAATGACCAACGAACTATCTCCT	GAATGACCAACGAACTATCTCCT
CL2-32033524	52.05	TTGAAGCAAGACTTGGGACTTA	AATATAGCATACTGAATCCAGGAGG
CL2-32288116	50.1	ATGGAAGTTTTAAATTGATACAATTATG	GTATCAATTTAAATCTAGAATTTTGAAGTG
CL2-32316840	53.6	GATCAAGTCATATCAAATCAATTCATTATTTG	CAAATATGACGTCAGAAGTAACTTGAC
CL2-32330086	50.7	GCATATATTGAAGAACTCCATCTTAC	GTGCTCAAATTGAAATAGTTTATGG
CL2-35629490	46.8	GTTTGAACTGAGGAAGACTT	CCCTACAGAATCCCATCAA
