# Supplementary material for: Molecular Markers and Marker-Assisted Selection Provide Genetic Insights for Identifying Key Quantitative Trait Locus for Watermelon Rind Thickness
Source: Int J Mol Sci. 2024 Sep 26;25(19):10341. doi: 10.3390/ijms251910341 (PMC11477180; doi:10.3390/ijms251910341)
Supplement: Supplementary file 1 [file ijms-25-10341-s001.zip › Supplementary Table S5.pdf]

Supplementary Table S5. Information on KASP markers based on 97103 v2 reference genome on chromosome 2

Position	primer_X	primer_Y	primer_C
CL2-32335621	GAAGGTGACCAAGTTCATGCTCAATTTTGTCTCTTCGTAACTGAAAATTTC	GAAGGTCGGAGTCAACGGATTCAATTTTGTCTCTTCGTAACTGAAAATTTG	GCCTTAACCTGCATATAATTGATGCATC
CL2-32346717	GAAGGTGACCAAGTTCATGCTGAGACTCAAATTGAAACAAAACTCAAACT	GAAGGTCGGAGTCAACGGATTAGACTCAAATTGAAACAAAACTCAAACC	TAGACCCTAAGTTCTAAAATATTACAATTTTACC
CL2-32349047	GAAGGTGACCAAGTTCATGCTGGGGATTTAGATTTCAGCCACG	GAAGGTCGGAGTCAACGGATTGGGGATTTAGATTTCAGCCACC	GGGAAGAGGCAGATCCTTCTCCA
CL2-32358427	GAAGGTGACCAAGTTCATGCTGTGGACCTATTAACCCACCAAACA	GAAGGTCGGAGTCAACGGATTTGGACCTATTAACCCACCAAACG	GATGCGTCTATTAATACCATAAAATATCTAAATG
CL2-33079009	GAAGGTGACCAAGTTCATGCTCACTAAAGACTAAAAGTGAGTGTTTAGTA	GAAGGTCGGAGTCAACGGATTACTAAAGACTAAAAGTGAGTGTTTAGTG	CCCTAGATTTTATGATGTACACTCTTAACG
CL2-33429760	GAAGGTGACCAAGTTCATGCTACTTACTGTGGAGATGCTCAAAGAA	GAAGGTCGGAGTCAACGGATTCTTACTGTGGAGATGCTCAAAGAG	GGATGATGGTTTTTGAATATGCTCCAAATG
