# Supplementary figures and images for: Molecular Markers and Marker-Assisted Selection Provide Genetic Insights for Identifying Key Quantitative Trait Locus for Watermelon Rind Thickness
Source: Int J Mol Sci. 2024 Sep 26;25(19):10341. doi: 10.3390/ijms251910341 (PMC11477180; doi:10.3390/ijms251910341)

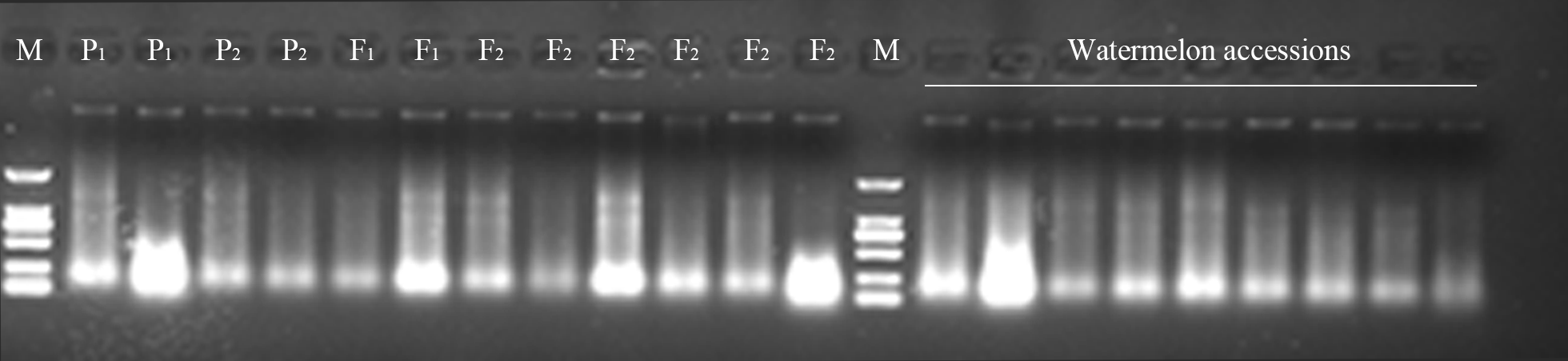

Supplement: Supplementary file 1 [file ijms-25-10341-s001.zip › Supplementary Figure S1.tif]

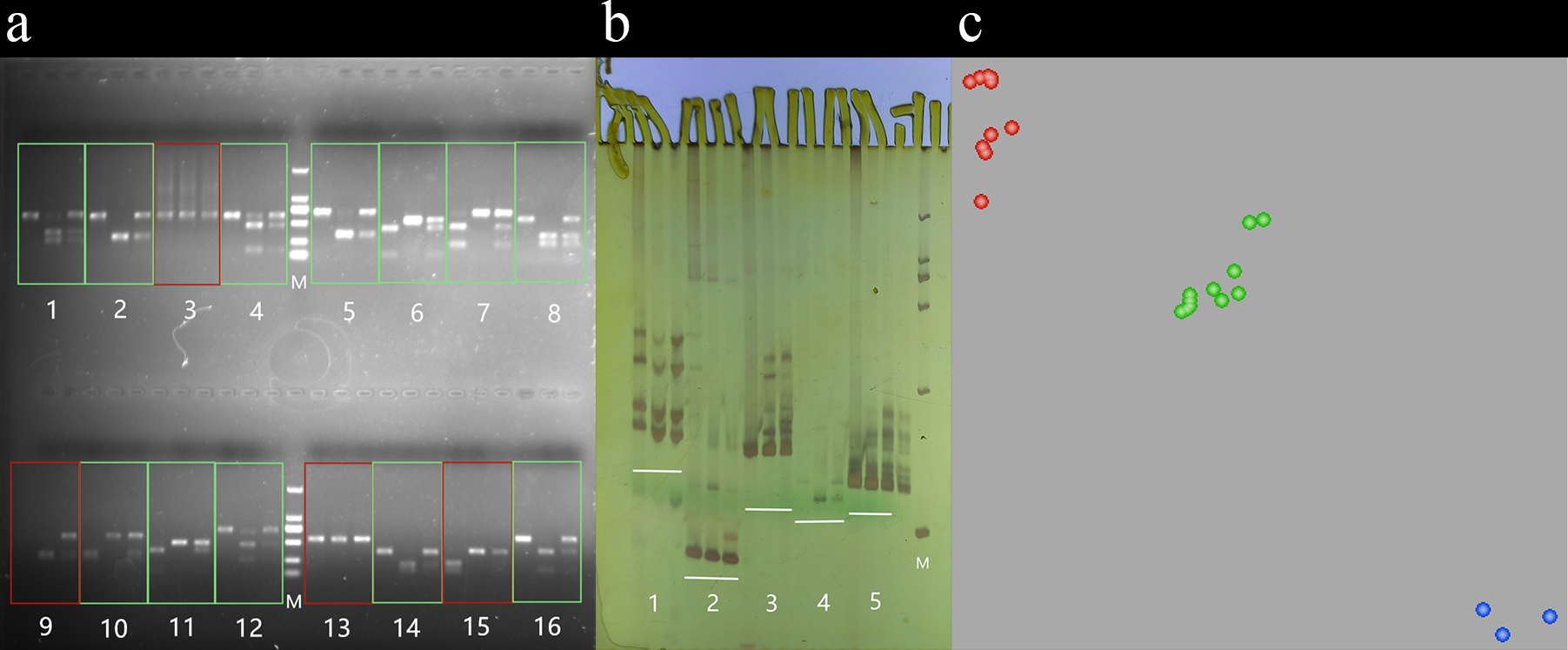

Supplement: Supplementary file 1 [file ijms-25-10341-s001.zip › Supplementary Figure S2.tif]

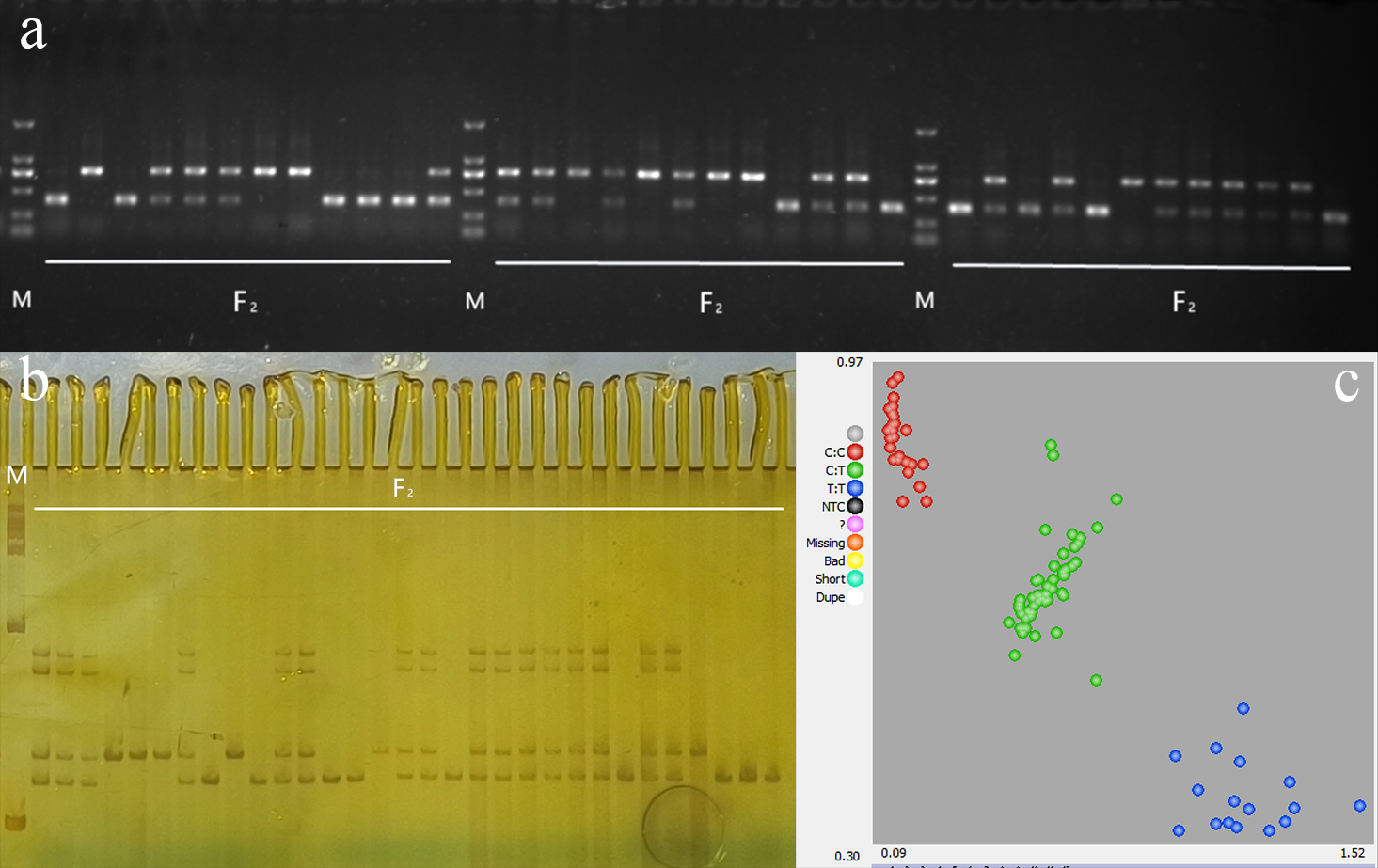

Supplement: Supplementary file 1 [file ijms-25-10341-s001.zip › Supplementary Figure S3.tif]

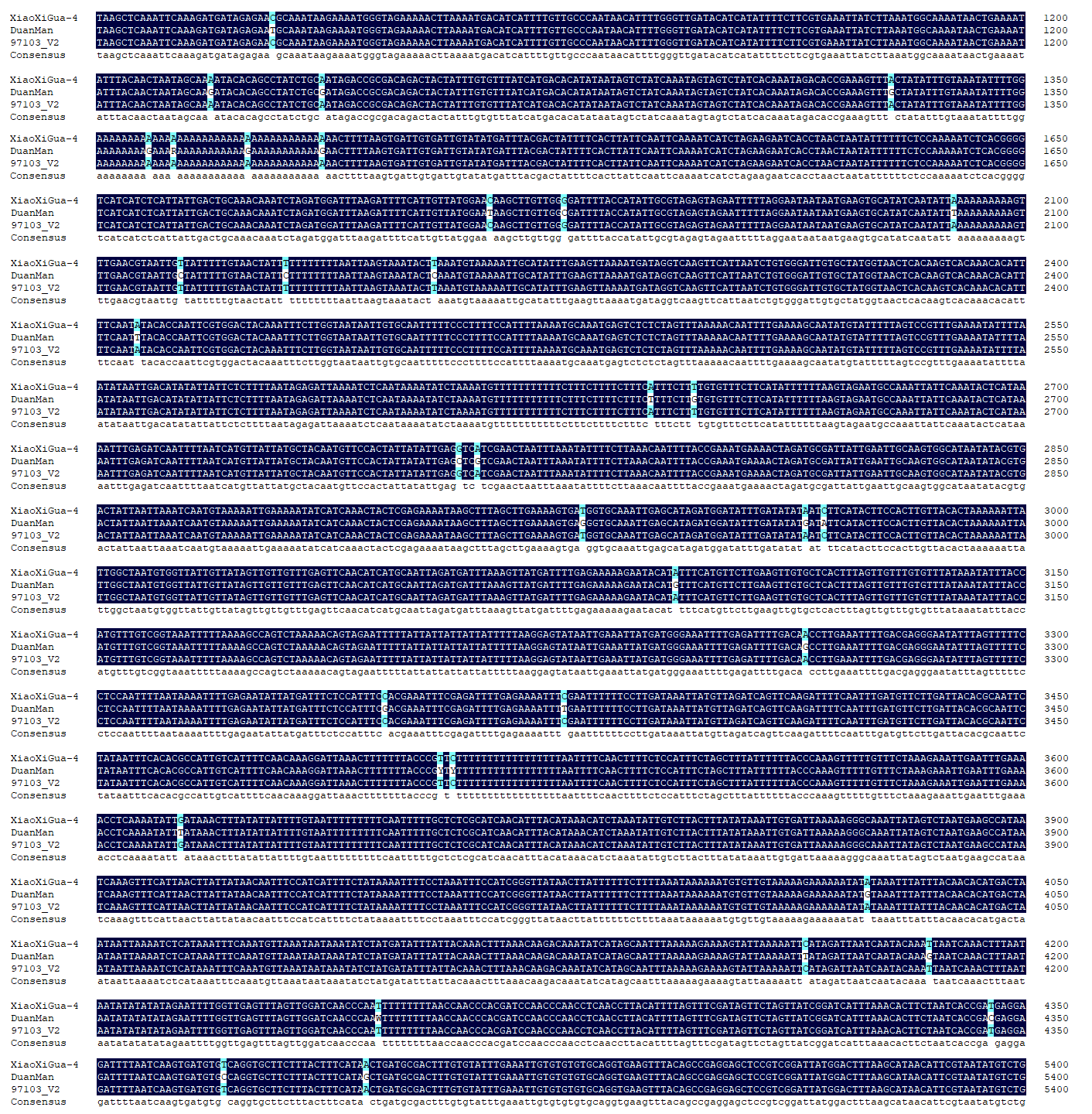

Supplement: Supplementary file 1 [file ijms-25-10341-s001.zip › Supplementary Figure S4.tif]

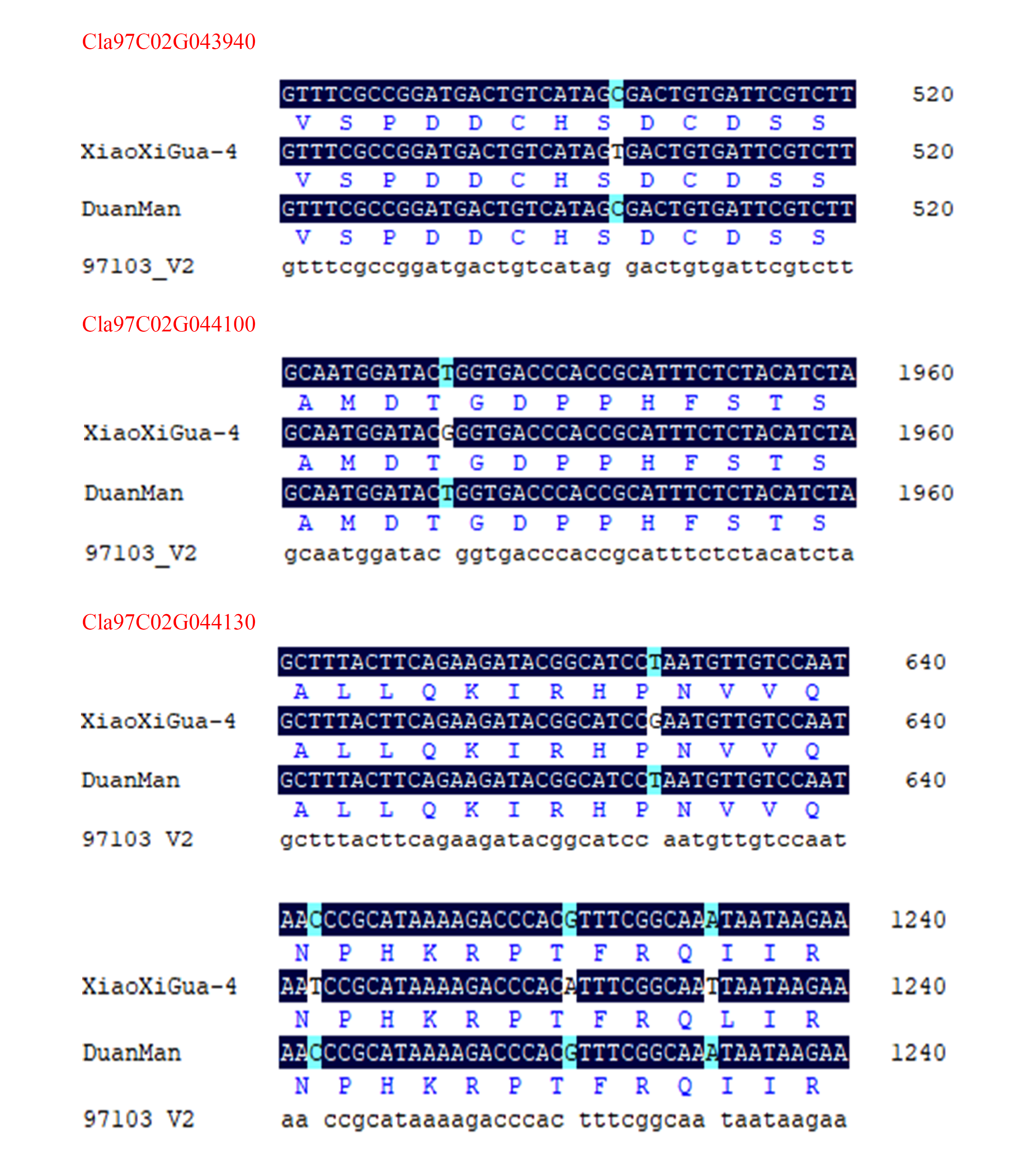

Supplement: Supplementary file 1 [file ijms-25-10341-s001.zip › Supplementary Figure S5.tif]
